# What Silly Postures Tell Us about the Brain

**DOI:** 10.3389/fnins.2012.00154

**Published:** 2012-10-11

**Authors:** Samuel J. Sober, Konrad P. Körding

**Affiliations:** ^1^Department of Biology, Emory UniversityAtlanta, GA, USA; ^2^Department of Physical Medicine and Rehabilitation, Northwestern UniversityChicago, IL, USA; ^3^Department of Physiology, Northwestern UniversityChicago, IL, USA; ^4^Department of Applied Mathematics, Northwestern UniversityChicago, IL, USA; ^5^The Rehabilitation Institute of ChicagoChicago, IL, USA

Because it looks funny and is pretty useless, we do not frequently touch our shoulder with our ear. It turns out that this insight has led to an exciting discovery in the field of cue combination. To interpret our senses, we rely on other senses. For example, to know how the image on our retina relates to the outside world, we need to know the alignment of our eyeball with respect to the outside world. To know this alignment, in turn, we need to know the orientation of our head relative to the outside world. Similar problems occur in other sensory and motor contexts. For example, the perceived direction of sound depends on the orientation of our head and movements of our body rely on the alignment of our body relative to the outside world. One way of formalizing these problems uses the idea of a coordinate system: every sensor provides information in a particular coordinates (e.g., retinal coordinates for visual input or joint angle coordinates for proprioceptive information).

The fact that sensory information arrives in many different coordinate systems produces a challenge for the brain: how to combine information that is encoded in different coordinate systems for successful movements. Both behavioral (Soechting and Flanders, 1989; van Beers et al., 1999; Sober and Sabes, 2003) and neural recording (Wallace et al., 1998; Fetsch et al., 2007) studies have explored how the brain “transforms” information across coordinate systems. It is generally understood that information degrades as it is transformed across coordinate systems.

Another important fact about information integration is that the brain must make all of its decisions based on unreliable and sometimes conflicting sensory data. Crucially, sensory uncertainty can be reduced by combining information from multiple sensors. Any combination needs to assign weights to different inputs. Should each sensor have the same importance or are some sensors more important than others? Based on studies showing that the brain often weights sensory inputs in a near-optimal fashion (Yuille and Kersten, 2006; Trommershauser et al., 2010), it is generally understood that the brain is exquisitely sensitive to statistical issues that occur when combining information.

That coordinate transforms are costly and sensory information is uncertain sets the stage for a remarkably interesting problem. Tasks require precision in the relevant coordinate system. Imprecise sensory information arrives from multiple sources. How should the brain combine information from multiple sources to move successfully? One intuition is that information that requires less coordinate transformation should be trusted more than information that requires more transformation – it should get more weight. The current study by Blohm and Burns (2010) tests a crucial prediction of this intuition: if one sensor is made more difficult to transform into the required coordinate system then it should be assigned a lower weight.

The authors begin with an established cue-conflict paradigm that allows the experimenter to measure the weight subjects put on vision versus proprioception (Sober and Sabes, 2003, 2005). Then, in a variant of the experiment, the subjects performed the task while holding their heads at a 30° angle. The rationale was that in this very unusual posture, we should have more uncertainty about the alignment of the head-centric and the body-centric coordinate systems. If this increases the difficulty of coordinate transformations then subjects should show less reliance on signals that must be transformed across coordinate systems.

The authors beautifully show that changing the difficulty of the transformation affects weights. For example, in the experiment, proprioceptive information about the hand relative to the body should be independent of head tilt. Visual information about the same variable, on the other hand, should be affected by the tilt as it requires converting information from retinal to joint coordinates. Indeed, the authors find that head tilt leads to smaller visual weights (see Figure 8A, light blue line). However, at some level the paper raises as many questions as it answers, since there seem to be two completely independent (but not mutually exclusive) interpretations of the results.

One interpretation, provided by the authors, is that the precision of our sense of head rotation is probably good when the head is held normally and less good when the head is rotated. This should be true due to the Weber-law properties of many sensors, which can result from the signal-dependent noise that has been characterized in the relevant orientation sensors. In this case, it is uncertainty about the relative orientation of the head to the body that gives rise on uncertainty about the alignment of the coordinate systems (Kording and Tenenbaum, 2006). In this interpretation transformed signals are given less weight during head rotation simply because our position sense from the eyes is made less reliable because of uncertainty about the head position.

Another interpretation is that the precision of computations in the nervous system should be better for coordinate transformations that happen more often. As the head is usually roughly at a 90° angle relative to the shoulders, the nervous system has more experience and might devotes more neural resources to the associated coordinate transformation, independent of any signal-dependent noise arising from peripheral sensors. In this interpretation transformed signals are given less weight because the brain avoids the neural noise associated with performing a less-familiar version of the transformation.

The paper thus opens up a new set of questions that should also be asked about previous studies (Sober and Sabes, 2003, 2005). How much of cross-modal weighting is determined by neural noise in coordinate transfers and how much is due to uncertainty about the coordinate system alignments? Future variants of the experiment could give additional cues about the relative alignment of the head or remove such cues. Such a manipulation should have no effect if neural noise dominates and should have a strong effect if alignment uncertainty dominates.
